# 

**Published:** 1895-11

**Authors:** 


### CONTENTS.

ORIGINAL ARTICLES:
- Gastritis Caninus (Non-Specific). By CECIL FRENCH, D.V.S. . . . . . 697
- Immobillty in the Treatment of Diseases of Extremities. By W. L. WILLIAMS, V.S. 703
- President's Address before the U. S. V. M. A. By W. HORACE HOSKINS, D.V.S. 708
- Physical Diagnosis. By JACOB HELMER, D.V.S. . . . . . . . . . 711
- Plural Pregnancy, Involving a Question as to Sire. By DR. W. H. HARBAUGH . 717
- Enteralgia. By L. E. WILLYOUNG, D.V.S. . . . . . . . . . . 721
- Hygiene. By DR. WILSON HUFF . . . . . . . . . . . 726

U. S. V. M. A. . . . . . . . . . . . . . . . . . . 730

CORRESPONDENCE:
- Reminiscences of the Intelligence of Animals. By ISAIAH MICHENER, V.S. . 731
- Stable Hygiene. By W. R. CLAUSSEN, V.S. . . . . . . . . . . 733
- The Rudolph Leuckart Celebration. By CHAS. WARDELL STILES . . . . 735

LEGISLATION:
- New York. By JOS. N. B. RAWLE, L.B., M.D.S., V.S. . . . . . . . 737
- Pennsylvania State Board of Veterinary Examiners . . . . . . . 739

REPORTS OF CASES:
- Enterotomy on a Bitch. By CECIL FRENCH, D.V.S. . . . . . . . 740
- Recto-Vaginal Fistula. By A. JASME, V.S. . . . . . . . . . 741
- Foreign Matter in Alimentary Canal. By JUDSON BLACK, V.S. . . . . . 742
- An Interesting Case. By D. W. GILBERT, V.S. . . . . . . . . . 743
- Value of Tips in Shoeing. By CHAS. BLAND, V.S. . . . . . . . . 743

CONTROL WORK . . . . . . . . . . . . . . . . . 744

EDITORIALS:
- And Now Pennsylvania—The Attitude of the Massachusetts Farmers' League—Prof. Theobald Smith—Laymen in the Department of Agriculture in England 746–748

OBITUARY—Pasteur . . . . . . . . . . . . . . . 748

EXAMINATION QUESTIONS:
- Veterinary Examinations, State of New York . . . . . . . . . . 752
- Civil Service Examination, New York City . . . . . . . . . . 755
- Candidates' Examination, State of Ohio . . . . . . . . . . . 755

SOCIETY MEETINGS AND PROCEEDINGS:
- New York State Veterinary Medical Society—Keystone Veterinary Medical Association—Iowa State Veterinary Medical Association—Montreal Veterinary Medical Association—Veterinary Medical Association of New Jersey . . 757–769

AMONG THE COLLEGES:
- University of California—Harvard—Ohio State University—British Schools . 769–771

Report of Army Committee. U. S. V. M. A. By DR. J. P. TURNER . . . 771

AMONG PEOPLE AND THEIR DOINGS—NEW INVENTIONS—PERSONALS . . 773–780

